# Arrhythmogenic Cardiomyopathy: One, None and a Hundred Thousand Diseases

**DOI:** 10.3390/jpm12081256

**Published:** 2022-07-30

**Authors:** Giovanni Peretto, Patrizio Mazzone

**Affiliations:** 1 Department of Cardiac Electrophysiology and Arrhythmology, IRCCS San Raffaele Scientific Institute, 20132 Milan, Italy; mazzone.patrizio@hsr.it; 2 School of Medicine, Vita-Salute San Raffaele University, 20132 Milan, Italy

According to the most recent expert consensus statement, arrhythmogenic cardiomyopathy (AC) is defined as an arrhythmogenic heart muscle disorder, not explained by ischemic, hypertensive, or valvular heart disease, presenting clinically as symptoms or documentation of atrial fibrillation, conduction disease, and/or right ventricular (RV) and/or left ventricular (LV) arrhythmia [1]. In daily clinical practice, AC mainly refers to a spectrum of nonischemic cardiomyopathies of either genetic or nongenetic etiology, capable of increasing the risk of sudden cardiac death (SCD) from malignant ventricular arrhythmia (VA).

In its original definition [2], AC indicated a specific disease, also known as arrhythmogenic right ventricular dysplasia (ARVD), with defined dystrophic features at histopathology and selectively involving the RV. However, following improved knowledge and characterization of the disease, the biventricular and left dominant phenotypes have been subsequently described [1]. Remarkably, the inclusion of isolated LV involvement in AC has led to a huge amplification of differential diagnoses, as also reported in the recent Padua criteria [3]. In fact, even when overt hypertrophic and dilated cardiomyopathy phenotypes have been ruled out, a broad range of genetic and acquired cardiomyopathies associated with VA display overlapping features with LVAC. In this setting, genotyping has been proposed as the cornerstone for differential diagnosis [3]. Nonetheless, even in the absence of a defined etiology, an increased incidence of VA has been reported in patients with a nonischemic LV scar [4], defined by a subepicardial/midwall stria pattern of late gadolinium enhancement (LGE) at cardiac magnetic resonance (CMR). Given the association between LV nonischemic substrate and documented VA, could we consider that as a form of LVAC? According to some authors [5], dilated cardiomyopathy constitutes an overlapping phenotype with AC rather than a true differential diagnosis. In fact, mutations in specific genes have been associated both with AC and dilated cardiomyopathy [1,5], and VA have been reported to occur before the onset of an overt LV dilation and dysfunction. For instance, age-related penetrance has been described in specific genetic cardiomyopathies such as those associated with mutations in the LMNA gene [6].

Among the newly described phenotypes consistent with the AC spectrum, left ventricular noncompaction (LVNC) and arrhythmogenic mitral valve prolapse (AMVP) are currently under investigation [7,8]. For instance, inflammation and myocardial wall fibrosis have been described in AMVP in addition to ectopy-triggered malignant VA [9]. For both diseases, the structural or scar-related component of arrhythmogenesis still remains to be elucidated. As for the acquired diseases, myocarditis represents a major cause of VA [10]. Since genetic myocarditis has been described as well [11], the role of myocardial inflammation as a driver for VA still needs to be investigated in primary cardiomyopathies [12] in light of the possible therapeutic implications [13]. Last but not least, the role of comorbidities potentially related to arrhythmias, such as thyroid dysfunction [14], anemia [15], neuromuscular [16], and systemic rheumatologic diseases [17], are likely underreported. Beyond VA, bradyarrhythmia and atrial tachyarrhythmia complicate many diseases of the AC spectrum, including cardiac sarcoidosis, giant cell myocarditis, and LMNA cardiomyopathy [6,10].

In recent years, a significant improvement in diagnostic techniques has occurred, allowing for the earlier detection of both cardiomyopathy signs and arrhythmic events. In particular, CMR has shown a major role in identifying abnormal substrates even in the absence of overt cardiomyopathy phenotypes [18]. In parallel, the advent of next-generation sequencing has led to a significant improvement in the detection of genetic abnormalities in patients with undefined cardiomyopathies [3]. Among other techniques, nuclear medicine imaging and endomyocardial biopsy allow identification of myocardial inflammation [19,20], whereas delayed enhanced CT scan offers an alternative to CMR for nonischemic substrate identification in cardiac device carriers [21]. Finally, three-dimensional electroanatomical maps are nowadays capable of identifying electrical abnormalities consistent with the early stages of nonischemic cardiomyopathies [22]. On the other hand, the application of continuous electrical monitoring tools has significantly improved the detection of arrhythmic events playing a defined prognostic role and/or constituting specific treatment targets [23].

As for the prognostic assessment, innovative and dedicated tools are nowadays available for arrhythmic risk stratification as a result of a multiparametric integration of prognostic factors. This applies to special AC etiologies such as LMNA cardiomyopathy [24,25]. For other undefined diseases, risk factors still need to be defined. In this context, it should be noted that the likelihood of arrhythmic events may be estimated even when the specific etiology of AC is unknown. Similar considerations apply to left ventricular ejection fraction (LVEF) in patients diagnosed with nonischemic cardiomyopathy without further characterization [18]. However, in the specific case of AC, the usefulness of LVEF is limited for SCD estimation since the systolic function is preserved or only mildly reduced in most patients. Subsequently, earlier prognosticators should be investigated by means of advanced and multimodal diagnostic workup [26]. For instance, septal localization of the scar has been associated with worse outcomes even in undefined nonischemic cardiomyopathies [22,27]. Improvement in available tools for risk stratification are advocated to guide patient selection for a primary prevention ICD implant. Programmed ventricular stimulation may offer guidance in restricted cases [28].

A number of therapeutic strategies directed to the AC spectrum are currently available, ranging from conservative pharmacological regimens to interventional approaches, namely cardiac device implant and catheter ablation of arrhythmia. Among ablation techniques, bipolar radiofrequency delivery may significantly improve the capability of reaching deep intramural substrates [29], especially in patients with midwall nonischemic scar [30]. Last but not least, preclinical studies have shown plenty of novel arrhythmogenic mechanisms involving specific genetic and acquired cardiomyopathies, allowing the identification of new promising targets for molecular treatment [31,32]. In this setting, multidisciplinary management offers a valuable improvement and fosters individualized patient care [16,33].

To address many of these topics, we are launching a Special Issue in the *Journal of Personalized Medicine* entitled “Progress in Pathogenesis, Diagnosis and Treatment of Cardiac Arrhythmia in Cardiomyopathies”. We are confident that many brilliant scientists involved in this emerging research field will valuably contribute to this Special Issue.

## Data Availability

Not applicable.
